# Partial paternal uniparental disomy (UPD) of chromosome 1 in a patient with Stargardt disease

**Published:** 2007-01-26

**Authors:** R. Riveiro-Alvarez, D. Valverde, I. Lorda-Sanchez, M. J. Trujillo-Tiebas, D. Cantalapiedra, E. Vallespin, J. Aguirre-Lamban, C. Ramos, C. Ayuso

**Affiliations:** Fundacion Jimenez Diaz, Genetics, Reyes Catolicos 2, Madrid, Madrid, Spain

## Abstract

**Purpose:**

Stargardt disease (STGD) is the most common juvenile macular dystrophy, characterized by central visual impairment. All recessively inherited cases are thought to be due to mutations in the *ABCA4* gene, mapped to 1p21-p13.

**Methods:**

To describe a form of non-mendelian inheritance in a patient with STGD identified through the course of a conventional mutational screening performed on 77 STGD families. DNA from the patient and relatives was analyzed for variants in all 50 exons of the *ABCA4* gene by screening on the ABCR400 microarray; results were confirmed by direct sequencing. Haplotype analyses, standard and high-resolution (HR) karyotypes, and multiplex ligation-dependent probe amplification (MLPA) were also performed.

**Results:**

A patient with STGD caused by the homozygous p.Arg1129Leu mutation in the *ABCA4* gene was found to be the daughter of a noncarrier mother and a father who was heterozygous for this change. Haplotype analysis suggested that no maternal *ABCA4* allele was transmitted to the patient. Microsatellite markers spanning the entire chromosome 1 identified a homozygous region of at least 4.4 Mb, involving the *ABCA4* gene. The cytogenetic study revealed normal female karyotype. Further evaluation with MLPA showed the patient had a normal dosage for both copies of the *ABCA4* gene, thus suggesting partial paternal isodisomy but not a maternal microdeletion.

**Conclusions:**

We report that recessive STGD can rarely be inherited from only one unaffected carrier parent in a non-mendelian manner. This study also demonstrates that genomic alterations contribute to only a small fraction of disease-associated alleles for *ABCA4*.

## Introduction

Stargardt disease (STGD1, MIM number 248200) is the most common hereditary macular dystrophy affecting children, with a prevalence of approximately 1:10000 [1]. It is characterized by central visual loss, atrophy of the retinal pigment epithelium (RPE) resembling a "beaten-bronze appearance" and the distribution of orange yellow flecks around the macula as well as midperiphery of the retina [2].

STGD is predominantly inherited as an autosomal recessive trait, although an autosomal dominant form has been also described [3].

The first genetic locus for recessive STGD was mapped to the short arm of chromosome 1 (1p21-p13) [4]. Mutations in *ABCA4* have been associated with autosomal recessive STGD (arSTGD) [5], autosomal recessive retinitis pigmentosa (arRP) [6], autosomal recessive cone-rod dystrophy (arCRD) [7] and age-related macular degeneration (AMD) [8].

Up to now, more than 400 disease-causing mutations have been identified in *ABCA4*. The mutation spectrum ranges from single base substitutions to deletions of several exons, although the majority of reported changes are missense mutations (ABCA4). As it has been described, mutations in *ABCA4* account for 66-80% of STGD-associated chromosomes [9,10].

Uniparental disomy (UPD) arises when a diploid individual carries both homologs of a chromosomal pair from a single parent (uniparental heterodisomy) or two copies of a single parental chromosome (uniparental isodisomy) [11]. Possible mechanisms of UPD include gamete complementation, trisomic zygote rescue (reduction to trisomy), postzygotic monosomy duplication, and somatic crossing-over [12,13].

The aim of this study is to describe, for the first time, a partial uniparental isodisomy in chromosome 1 including the *ABCA4* gene region, resulting in a STGD phenotype in the patient.

## Methods

### Ascertainment of patients

This molecular study was reviewed and approved by the Ethics Committee of the Hospital (Fundacion Jimenez Diaz). The recruitment of patients and relatives was carried out through six research groups that work in the retinal dystrophy Spanish network (EsRetNet). This study was performed according to the tenets of the Declaration of Helsinki and further reviews (Edinburgh, 2000).

### Mutation detection

Peripheral blood samples were taken, and genomic DNA was extracted using an automated DNA extractor (BioRobot EZ1, Qiagen, Hilden, Germany).

All exons of the *ABCA4* gene were analyzed for variants on the ABCR400 microarray, following a procedure described by Jaakson et al. [14]. The 50 exons of the ABCA4 gene, including the intron-exon junctions, were amplified by PCR primers as previously described [15], in order to confirm the results obtained from the microarray. These fragments were electrophoresed in a 3% agarose gel and purified using a DNA extraction kit (QIA-quick Gel Extraction Kit, Qiagen).

Sequencing reactions were performed using the 4-dye terminator cycle sequencing ready reaction kit (dRhodamine DNA Sequencing Kit, Applied Biosystems, Foster City, CA). Sequence products were purified through fine columns (Sephadex G-501, Princetown Separations, Adelphia, NJ) and resolved in an ABI Prism 3100 (Applied Biosystems).

### Haplotype analysis

Haplotypes were constructed using five microsatellite markers flanking the *ABCA4* gene (TEL-*D1S435*-*D1S2804*-*D1S2868*-*ABCA4*-*D1S236*-*D1S2664* -CEN).

For this case an extended haplotype was necessary; therefore 32 highly polymorphic markers spanning all chromosome 1 were selected from both of the NCBI-UniSTS website and the Linkage Mapping Set v2.5 Kit (Applied Byosistems). Microsatellite markers on chromosomes 13, 18, 21, X, and Y were also analyzed to confirm the biological relationship between the different members. After their amplification by PCR, fluorescently labeled products were mixed and electrophoresed on the ABI Prism 3100 (Applied Biosystems).

### Multiplex ligation-dependent probe amplification

The target sample was analyzed by Multiplex Ligation-dependent Probe Amplification (MLPA) and compared to five control DNAs selected from control population. MLPA reagents were obtained from MRC-Holland B.V. (SALSA MLPA KIT P151 and 152 ABCA4, Amsterdam, The Netherlands), and the reactions were performed according to the manufacturer's instructions (MRC-Holland).

### Cytogenetic analyses

From the STGD patient, we extracted a second blood sample for chromosome analysis, which was conducted using both standard and high resolution (HR) karyotyping methods (800-900 bands).

## Results

### Clinical findings

**Figure 1:**
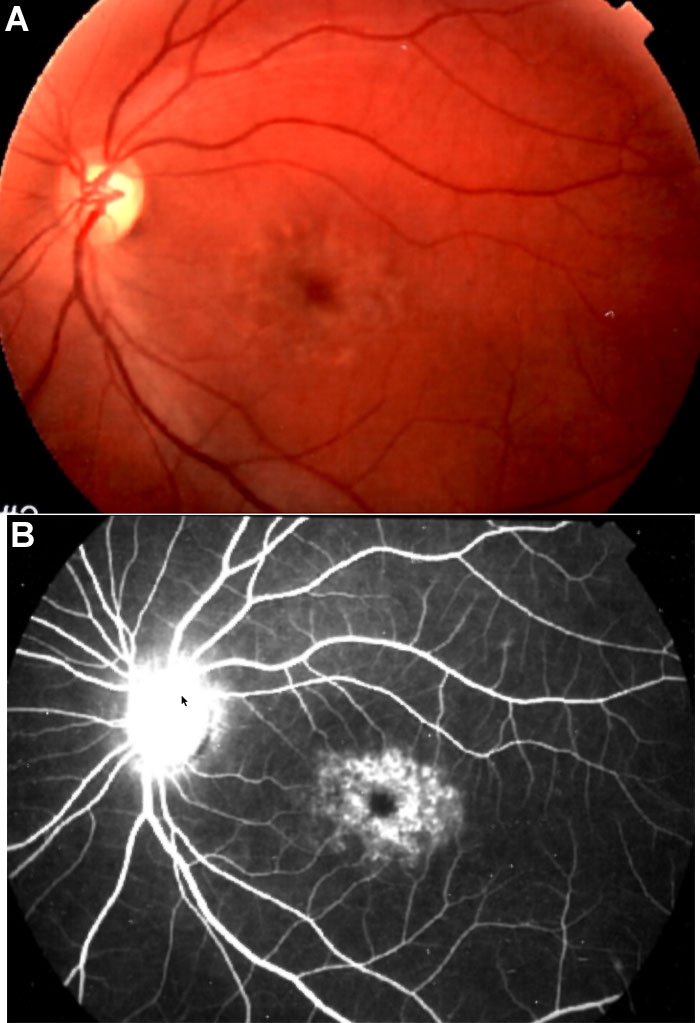
Ophthalmological findings. **A**: Fundus photograph of the patient's left eye shows macular yellow flecks characteristic of Stargardt macular dystrophy. **B**: Angiofluoresceingraphy (AFG) shows silent choroid and atrophic retinal pigment epithelium at the macular area.

The patient was a Caucasian female born to nonconsanguineous, healthy parents. Her mother was 34 years old at the time of delivery. Ophthalmic evaluation documented the characteristic findings of STGD: onset of her retinal disease occurred at the age of 2-3 years, with mild vision loss and strabismus. For a long time, photophobia and dyschromatopsia were the only presented symptoms. At the age of 35, her visual acuity was 100/200, biomicroscopy was normal and funduscopy revealed yellowish flecks at the macula (Figure 1A). Angiofluoresceingraphy (AFG) showed silent choroid areas and hyperfluorescent dots at the perifoveal retina, resembling little defects at the RPE (Figure 1B). The patient did not show any physical abnormalities, and she has a four-year-old asymptomatic daughter.

### Molecular and cytogenetics analyses

**Figure 2:**
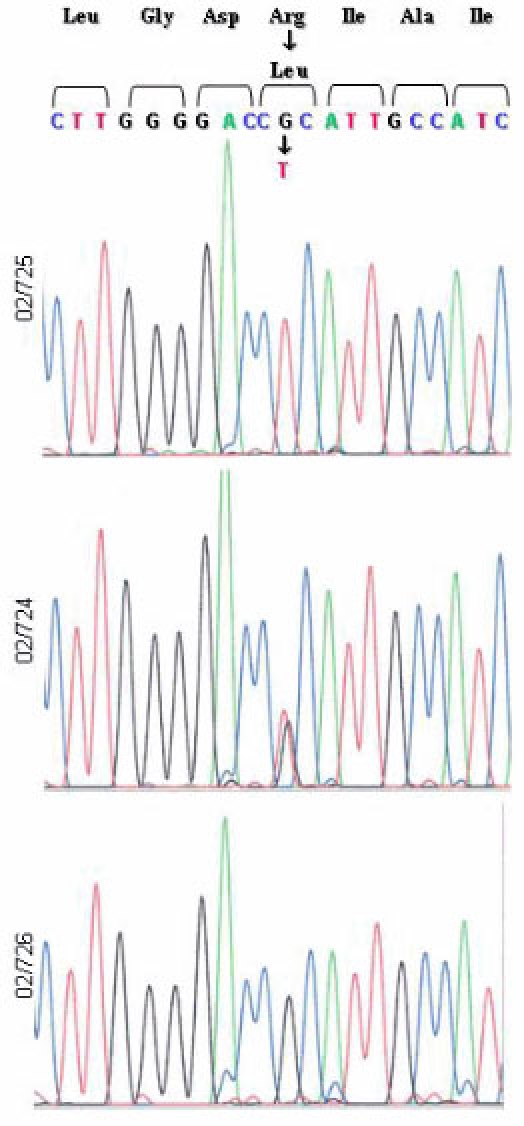
The DNA sequence of *ABCA4* codons 1126 to 1132, in the index patient and both parents. The patient (02/725) shows a homozygous pattern for the missense p.Arg1129Leu mutation. Her father (02/724) presents a heterozygous pattern, while her mother (02/726) shows a homozygous wild-type sequence.

In this molecular study, we identified a 40-year-old woman diagnosed with STGD in childhood, who had an apparently homozygous pattern for the missense p.Arg1129Leu (c.3386G>T) mutation. No other pathogenic mutation aside from two homozygous polymorphisms (p.His423Arg (c.1268A>G), IVS33+48 C>T) [16,17], were found in the screening of the gene using the ABCR400 microarray. Her brother and her sister, who were not affected, also carried this mutation heterozygously. The R1129L mutation was present in a heterozygous pattern in her unaffected father, but interestingly her mother did not harbor this change (Figure 2).

Closely linked microsatellite markers were analyzed (TEL-*D1S435*-*D1S2804*-*D1S2868*-*ABCA4*-*D1S236*-*D1S2664* -CEN). As expected, the patient was homozygous at all marker loci of one region of the short arm of chromosome 1, with the allele present at each locus identical to one of the alleles present in her father. The homozygous (isodisomic) region spanned a physical distance of 4.4 Mb, which included the *ABCA4* locus.

To elucidate the possibility that the homozygous p.Arg1129Leu variant was due to a deletion spanning the maternal *ABCA4* gene, we obtained standard and HR karyotypes from the patient. Both cytogenetic analyses showed normal female karyotype, 46, XX.

**Figure 3:**
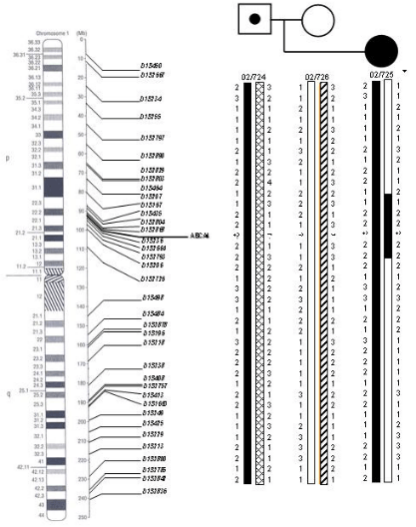
Pedigree showing the pattern of the 32 genetic markers used for chromosome 1 in the proband and in her parents. Patient's haplotype demonstrates that 5 markers for 1p, from *D1S435* to *D1S2664* (black bar), were homozygous and derived from one paternal chromosomal homolog. We concluded that paternal partial isodisomy was present, with breakpoints between 1p22.3 (*D1S167*-*D1S435*) and 1p22.1 (*D1S2664*-*D1S2793*). Markers *D1S2793* and *D1S206* could also be involved in the isodisomy, but were not informative (striped bar). Markers located on the short arm (excluding the isodisomic region), on the centromeric region, and on the distal region from the long arm (shown in brackets) clearly indicate normal maternal and paternal segregation. For the remaining markers along 1q, we could not distinguish between normal segregation or (partial) maternal heterodisomy. The location of the genetic markers, placed on the physical and cytogenetic maps of chromosome 1, was established according to the information contained in the NCBI database.

Further analyses of 32 informative microsatellite markers scattered along both arms of chromosome 1 were also performed. The patient presented biparental heterozygous markers near the centromere (*D1S2726*) and on the distal short (*D1S450*, *D1S2667*), and long (*D1S2785*) arms. For the remaining markers spanning both arms of chromosome 1, we could not distinguish between normal biparental segregation or maternal uniparental heterodisomy (Figure 3).

The assumed biological relationship of the patient to her parents was confirmed by analyses of a number of microsatellite markers on chromosomes 13, 18, 21, X, and Y (data not shown).

**Figure 4:**
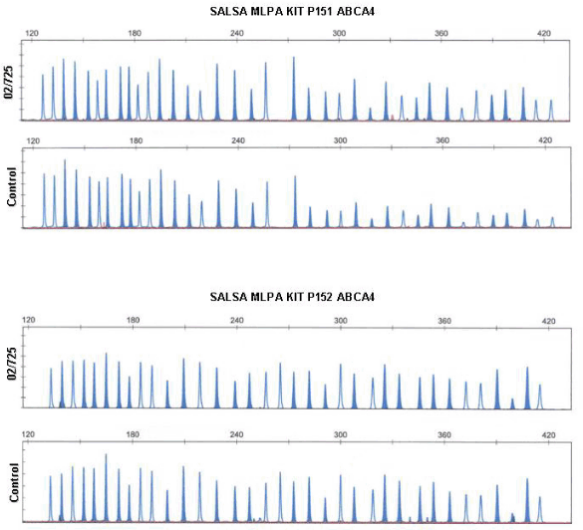
Multiplex ligation-dependent probe amplification (MLPA) of *ABCA4* exons. Multiplex ligation-dependent probe amplification (MLPA) electropherograms from the proband and one control sample. Shaded peaks represent *ABCA4* exon probes, normal peaks represent synthetic probes. Every probe presented a normal amplification pattern, suggesting normal dosage for both copies of *ABCA4*. Therefore, the possibility of a microdeletion was ruled out.

Finally, we assessed dosage for the *ABCA4* locus by using the novel MLPA technique. The index case was compared to five control samples and no deletion or duplication was detected for any of the 48 amplified probes. This result firmly confirmed that both *ABCA4* alleles were present in our patient (Figure 4).

## Discussion

In this work, we describe a patient presenting typical STGD due to mutations in the *ABCA4* gene. This gene encodes the *ABCA4* protein, a member of the ATP-binding cassette transporters superfamily. It is involved in the transport of vitamin A derivatives across the membrane of the outer segment discs of photoreceptors [18,19].

In this affected woman, the homozygous p.Arg1129Leu mutation was identified. The inheritance pattern of the disease-associated alleles was not autosomal recessive as usual. Heterozygosity for the mutation was only detected in her father. Assumed paternity was confirmed by different STR markers [20]; therefore the proband either had (partial) paternal isodisomy for chromosome 1 or an unbalanced karyotype due to maternal microdeletion involving chromosome 1p, leading to hemizygosity for the *ABCA4* locus. Both standard and HR karyotypes were normal, which excluded major structural chromosomal abnormalities. Dosage analysis performed by MLPA confirmed the presence of two copies of the *ABCA4* gene, pointing to partial paternal isodisomy.

The course and severity of the proband's visual disorder fit well within the range of clinical phenotypes exhibited by other reported STGD patients carrying the homozygous p.Arg1129Leu mutation. This, in turn, supports that this disease-associated allele conveys a moderate severity in the pathogenesis of STGD. This is the most frequently occurring mutation in this gene among Spanish population, comprising about 24% of all reported *ABCA4* alleles [21].

The molecular genetics findings in the patient indicate that she inherited both copies of chromosome 1, one from each progenitor, except for the paternal isodisomic region found on the short arm of the maternal homolog. Therefore, in this patient the recombination has to be postzygotic (mitotic). This could be due to recombination in a trisomic cell, followed by trisomy rescue or loss of maternal chromosome 1p followed by reduplication of paternal chromosome 1p. Postzygotic recombination in a diploid cell can result in mosaicism with partial isodisomy; this mechanism could not be assessed because we were not able to test other tissues than peripheral lymphocytes, as any possible contact with this family was lost.

It is less likely that trisomy rescue occurs, since trisomies for chromosome 1 are extremely rare and these fetuses are thought to die before implantation [22]. If trisomy rescue was the mechanism, it must have occurred almost during the first cell division of the zygote, resulting in complete loss of trisomic cells.

We have also identified one particular region at 1q, where we could not distinguish between normal (biparental) segregation or maternal heterodisomy (Figure 2). We suggest that this latter mechanism is less likely to happen. Therefore this situation may be due to both parents sharing one of the two alleles at these markers. Maybe these are the most frequent alleles for these microsatellite markers among Spanish population, although heterozygosity for these markers may be higher among other populations.

Other examples of uniparental disomy of chromosome 1 have been reported, being of either paternal [23-26] or maternal origin [27-29]. Of these, two previously reported cases had retinal degeneration; one patient presented complete uniparental isodisomy leading to homozygosity for a splice-site mutation in the RPE65 gene [24] while an other patient had uniparental heterodisomy, with partial isodisomy, leading to an homozygosity for a missense mutation in the USH2A gene [23]. In reference to ophthalmic diseases, cases with uniparental disomy involving other chromosomes have also been described [24,30].

As described by Engel, one of the consequences of UPD may be interference to genomic imprinting [11]. As in our case, patients with uniparentally derived chromosomes 1 were ascertained because of the evolution of their recessive diseases, showing no additional genetic abnormalities. As it has been previously described, there are no parentally imprinted genes on chromosome 1 that have a major effect on phenotype [24]. However, an associated increased risk for suffering from recessive disorders must be considered, thus families with recessive inherited retinal diseases can have affected offspring, homozygous for the same mutation, even if the carrier's spouse has wild-type alleles at the disease locus.

Further studies must be conducted specifically on those cases in which only one mutation has been found in order to establish the incidence and therefore the risk of such events.

A non-mendelian inheritance pattern for STGD disease has been previously reported by Rozet et al [31]. They described one case showing severe retinitis pigmentosa, which presented a homozygous splice mutation (c.1938-1 G>A) at the *ABCA4* locus, due to maternal deletion. However, no direct evidence for deletion of the *ABCA4* gene could be found, thus a possible (partial) UPD may also be suspected. For this situation, MLPA would become a suitable alternative for detecting whether a deletion had occurred or not.

To the best of our knowledge, partial isodisomy for chromosome 1p, as described in this report, has not been described before. Nevertheless, one case of complete UPD resulting in STGD has been recently described [32].

This finding has an important consequence for genetic counseling of this type of patients; because the recurrence risk for partial UPD is low, the risk for STGD in her siblings, her daughter and future offspring is negligible.

The present study, which forms part of the conventional mutational analysis performed on 77 Spanish STGD families, suggests that genomic alterations contribute to only a small fraction of disease-associated alleles of *ABCA4*. Up to now, this is the major structural abnormality encompassing this gene that has been identified (HGMD). For those cases where a homozygous mutation is found, it is recommended to perform haplotype analyses in order to discard a possible situation of UPD. Apart from the detection of the homozygous p.Arg1129Leu mutation, the information of the homozygosity for both intragenic polymorphisms provided by the ABCR400 chip, added to the suspicion of noncontribution of the maternal allele.
